# The effect of event impact on fear of missing out: the chain mediation effect of coping styles and anxiety

**DOI:** 10.3389/fpsyg.2024.1382440

**Published:** 2024-04-18

**Authors:** Bofeng He, Zhenjing Tan, Kaiying Lai, Boyu Qiu, Suiping Wang

**Affiliations:** ^1^Philosophy and Social Science Laboratory of Reading and Development in Children and Adolescents (South China Normal University), Ministry of Education, Guangzhou, China; ^2^School of Psychology, South China Normal University, Guangzhou, China; ^3^School of Health Management, Guangzhou Medical University, Guangzhou, China; ^4^School of Education, South China Normal University, Guangzhou, China; ^5^School of Public Health, Guangdong Medical University, Dongguan, China; ^6^Guangzhou Institute of Educational Research, Guangzhou, China

**Keywords:** impact of event, fear of missing out, coping styles, anxiety, self-regulation

## Abstract

The fear of missing out is a generalized anxiety stemming from the possibility of not being present at new events or advantageous situations of others. To explore potential mechanisms, a survey measuring the impact of event, coping style, anxiety, and fear of missing out was conducted with 1,014 college students (367 males and 647 females, aged 19–24 years). In addition, the study delved deeper into the dimensions of intrusion, avoidance, and hyperarousal concerning the impact of event, examining their roles in coping style, anxiety, and fear of missing out. Results showed that: (1) The impact of event could predict the fear of missing out positively. (2) A more positive coping style is negatively associated with anxiety. (3) A chain mediation effect of coping style and anxiety is observed in the path from hyperarousal and avoidance to the fear of missing out. (4) In contrast to the hyperarousal or avoidance, the path from intrusion to the fear of missing out is mediated by anxiety but not coping style. These findings motivate us to implement different intervention tactics for varying impacts of events.

## 1 Introduction

The contemporary world has seen a tremendous surge in the availability of information, thanks to the rapid progress in communication technologies. These technologies enable us to stay connected and informed about current developments and occurrences at all times and from any location. However, the ease of access to information also presents challenges, such as the fear of missing out on something beneficial, an investment opportunity, or the fear of peers out performing oneself. Such apprehensions result in anxiety. Researchers refer to this psychological feeling as the fear of missing out (FoMO). The FoMO is defined as a form of anxiety stemming from the fear of missing out on novel experiences or events advantageous to others (Przybylski et al., 2013). People are susceptible to experiencing the FoMO when they feel anxious about missing out on advantageous investment opportunities while stock market indices are on the rise. Additionally, they may experience the FoMO when they want to stay constantly updated on the people and events around them during significant occurrences. FoMO is not a minority experience. A study conducted by a Chinese organization revealed that 15.2% of participants reported experiencing a severe FoMO (Wen, 2016). Notably, men tend to experience this fear more strongly than women (Przybylski et al., 2013).

As a new psychological state that has emerged alongside technological developments, numerous researchers have explore phenomenon of FoMO. The FoMO has been found to be associated with unfavorable psychological experiences and problematic behaviors (Swan and Kendall, 2016; Zou et al., 2018; Reer et al., 2019; Elhai et al., 2021). Individuals who experience the FoMO may also experience emotional problems such as anxiety and depression (Beyens et al., 2016; Swan and Kendall, 2016; Oberst et al., 2017; Dhir et al., 2018). They tend to stay constantly connected to the outside world to reduce anxiety, typically resulting in prolonged and frequent use of social media. This often leads to problematic use of social media (Beyens et al., 2016; Casale et al., 2018; Franchina et al., 2018; Reer et al., 2019; Balta et al., 2020; Elhai et al., 2020a,b; Duman and Ozkara, 2021; Guazzini et al., 2022). Driven by their desire to stay connected and avoid missing any information, individuals suffering from the FoMO may even use their phones while driving (Przybylski et al., 2013) or developing alcoholism (Riordan et al., 2015; Abri, 2017).

While it is widely acknowledged that a strong association exists between the fear of missing out and negative psychological and problematic behaviors. a conclusive relationship between these variables has not been established conclusively (Akbari et al., 2021). Most current research has primarily focused on the psychological and behavioral problems arising from the FoMO (Riordan et al., 2015; Swan and Kendall, 2016; Franchina et al., 2018; Xie et al., 2018; Elhai et al., 2020a; Fitzgerald et al., 2023). However, the causes of FoMO remain unclear, and further investigation is necessary to explore its potential mechanisms.

### 1.1 Impact of event and FoMO

The FoMO is influenced by various factors including personality traits, psychological needs, and life events (Przybylski et al., 2013; Blackwell et al., 2017). In addition to these individual idiosyncratic factors, an important exogenous factor is the impact of event (Turkle, 2011). Impact of event refer to life events that affect one's mental health and have negative consequences (Brown et al., 1973). Individuals are affected to varying degrees by events in their lives on the dimensions of intrusion, avoidance, and hyperarousal (Zilberg et al., 1982; Wu and Chan, 2003; Motlagh, 2010). The intrusive factor refers to an individual experiencing recurring and intrusive memories, such as nightmares, after being affected by an event. This can negatively impact the individual unconsciously or cognitively. Meanwhile, avoidance behavior occurs when an individual adopts strategies to steer clear of events, emotions, or memories associated with the event, such as avoiding people, places, or media content. Following a traumatic event, individuals may also experience hyperarousal, which is characterized by heightened physiological and psychological states. Symptoms may include irritability, increased alertness, insomnia, and heightened sensitivity to stimuli related to the event.

Research has considered impact of event as a holistic entity, arguing that it can lead to mental health issues such as depression and anxiety (Flory and Yehuda, 2015; Geng et al., 2019). Sudden impact of event may significantly affect an individual's mental and physical health, resulting in exhaustion and potential mental health issues (Lazarus and Opton, 1966). These events, such as failing exams, interpersonal relationship problems, or major social events, can result in physical and mental problems, negative emotions like insomnia, anxiety, depression, and, in severe cases, suicidal behavior for a college student. Furthermore, studies have examined the impact of event on individual mental health from three distinct dimensions. For instance, a study of 357 college students who experienced the impact of event. The findings indicate that impact of event significantly predict suicidal ideation solely on the dimension of higharousal (Briere et al., 2015). Another study, which focused on Vietnam War veterans, revealed that only intrusion significantly predicts suicidal ideation (Bell and Nye, 2007). Therefore, segmenting event impact into distinct dimensions will aid in our deeper understanding of the underlining mechanism.

Studies have shown that the impact of event was linked to FoMO. The impact of event can cause stress, mood swings, and other emotional issues (Barlow, 2000; Garnefski et al., 2001; Hughes and Shin, 2011). To alleviate these emotions, individuals may turn to the Internet or social media frequently for a long time, which in turn increase the level of FoMO (Farahani et al., 2011; Billieux et al., 2015; Buglass et al., 2017). A study on people's mental health levels during the COVID-2019 pandemic also found that when the major event of COVID-2019 occurred, people were more likely to suffer from FoMO due to anxiety that they would miss out on information related to the pandemic (Casale and Flett, 2020; Koban et al., 2022).

FoMO can be considered a reflection of deficient self-regulation (Przybylski et al., 2013). Self-regulation theory is a process that involves the interaction of three processes: personal, behavioral, and environmental (Bandura and Cervone, 1986). It is a systematic approach by which individuals direct their cognitions, emotions, and behaviors toward achieving their goals (Zimmerman, 2000). During the process of self-regulation, individuals plan and cyclically adapt their self-generated thoughts, feelings, and actions to achieve personal goals. However, when faced with an impact of event, the cyclical adaptive process of self-regulation is disrupted, resulting in significant cognitive, affective, and behavioral stress. The defensive response described can be referred to as an ego-barrier strategy. This strategy is intended to protect the self, but ultimately it can restrict the individual's growth and impede their development (Pintrich et al., 2012).

Individuals who experience impaired self-regulation may be in a state of diffuse anxiety at the affective level. At the cognitive level, there is a need to reevaluate the event, leading to a constant follow-up on related information. At the behavioral level, this is often accompanied by maladaptive social media use in order to stay informed and avoid missing out on information. Therefore, the impact of event can lead to the development of FoMO. From this theoretical perspective, FoMO can be understood as a hindrance to self-regulation caused by situational or chronic unsatisfied psychological needs (Przybylski et al., 2013). Studies from sports, education and games have found that the satisfaction of basic needs is closely linked to proactive behavioral regulation (Ryan et al., 2000; Przybylski et al., 2009).

From the perspective of impaired self-regulation, the path from impact of event to FoMO occurs in the form of emotionally, cognitively, and behaviorally impeded regulation. Emotionally, both regulatory stress and regulatory failure bring about emotional problems and anxiety. Cognitively and behaviorally, cognitive appraisal and behavioral regulation adjust coping strategies and goals, which may lead to poor coping style when individuals are unable to cope with appropriate cognitive strategies and actions. Therefore, there may be two mediating mechanisms in the path from impact of event to FoMO: (1) Maladaptive coping style due to impaired regulation following life event shocks, which subsequently triggers FoMO; and (2) Emotional problems arise following impact of event due to impaired emotional regulation, which then triggers generalized anxiety, leading to FoMO.

### 1.2 Mediation effect of coping style in the path from impact of event to FoMO

An individual's interaction with the environment may result in a burden, and the cognitive and behavioral efforts taken to alleviate this burden by making conscious, purposeful, and flexible adjustments are coping style (Lazarus, 1974; Joffe and Bast, 1978). Based on the attributes of coping style, they can be categorized into positive and negative coping styles (Xie, 1998). For example, when faced with difficulties and stress, individuals may choose to cope with positive coping styles such as changing their thoughts and talking to people to confide in them about their troubles, or they may choose to cope with negative coping styles such as trying to forget about things through smoking and drinking, excessive use of social media, and passive waiting for things to get worse.

Several studies have pointed out that coping style play a mediating role in the path from the impact of event to negative psychological experiences (Wolfradt et al., 2003; Kasi et al., 2012; Mahmoud et al., 2012; Roohafza et al., 2014). Additionally, some studies have argued for a positive association between the impact of event and coping style (Cao and Si, 2010). When individuals cope with negative life events negatively, it exacerbates their negative emotions (Tu and Guo, 2011).

In terms of theoretical foundations, the coping process model proposed by Lazarus suggests that the coping of a life event goes through two processes: cognitive assessment and coping behavior (Lazarus and Folkman, 1984). When a life event strikes, the individual first assesses the event and then chooses a strategy to cope based on the resources he or she possesses. Therefore, the situational nature of the life event and the individual's cognitive base will affect the choice of the coping style. And the impact of event can also bring challenges to the self-regulatory system. When a life event suddenly brings an impact that exerts pressure on an individual's external environment, the cognitive system needs to seek coordination between the internal and external environments in order to cope with the event. When the regulatory system is overstressed by the impact of event, it can lead to negative coping style.

Likewise, when individuals face impact of event, they tend to stay in touch with the outside world and gather as much relevant information as possible through prolonged and frequent use of internet applications such as social media (Bright and Logan, 2018; Fang et al., 2020; Deniz, 2021). Individuals hope to fulfill their need to stay connected to the outside world in this way to alleviate their anxiety, and at the same time, they will often fear that they will not be able to consistently keep up with people and events in the outside world and feel worried and anxious (Franchina et al., 2018; Elhai et al., 2020b). This misuse of social media is a kind of negative coping style, so it can be inferred that a negative coping style at the impact of event leads to FoMO. It was also noted above that the impact of event can lead to negative coping style, therefore, there is ample empirical and theoretical evidence that the coping style has a mediating role in the path from the impact of event to FoMO.

### 1.3 Mediation effect of anxiety in the path from impact of event to FoMO

Anxiety is a negative emotion that individuals experience as a result of perceiving or anticipating a threat, an undesirable consequence, and usually manifests itself as inner distress, apprehension, and restlessness (Bell and Sciacca, 2020). This emotion is often accompanied by increased activity of the autonomic nervous system, such as the presence of a rapid heartbeat, deepened breathing, sweating, muscle tension, and other physiological responses (Barlow, 2000; Etkin and Wager, 2007). Anxiety may affect an individual's mood, thinking, and behaviors, and severe anxiety disorders can affect the quality of life. According to epidemiological studies, anxiety disorders have a worldwide prevalence ranging from 5% to 10% worldwide (Kessler et al., 2007).

The relationship between impact of event and anxiety has been examined. It has been noted that after major life events such as natural disasters, adolescents are prone to anxiety problems such as fear, tension, and somatization (Reijneveld et al., 2003; Groome and Soureti, 2004; Kar and Bastia, 2006). According to the susceptibility-stress model of anxiety, anxiety arises when internal factors (e.g., personality) and external environmental factors (e.g., impact of event) activate potential susceptibility factors, catalyzing the development of anxiety (Barlow, 2000; Mathews and MacLeod, 2005; Tang et al., 2008).

The impact of event can trigger anxiety, which in turn can contribute to the FoMO. Conceptually, FoMO is conceptually defined as being a diffuse form of anxiety and is even considered a subcategory of anxiety (Przybylski et al., 2013; Huan You et al., 2018). Individuals who suffer from FoMO experience diffuse anxiety due to the fear of missing meaningful experiences of others. They have a strong expectation of consistently paying attention and following up on what others are doing and are concerned about missing out on novel experiences that others have. Apart from studies that have linked FoMO to anxiety, little attention has been paid to the path from anxiety to FoMO. Anxiety itself may lead to a more serious FoMO. FoMO is the fear of regret, which arises from an individual's generalized worry and anxiety about missing out on a social interaction, anxiety is the main factor that triggers the FoMO. Thus, it can be assumed that anxiety has a mediating role in the pathway from impact of event to the FoMO.

### 1.4 Chain mediating role of coping style and anxiety

Maladaptive coping styles may further exacerbate anxiety. It has been suggested that positive coping styles are protective factors for anxiety, whereas negative coping styles, such as avoidance, are risk factors for anxiety. Previous studies have shown that negative coping styles are positively correlated with anxiety levels (Kozora et al., 2005; Murberg and Bru, 2005; Lewis et al., 2012; Roohafza et al., 2014). In a study of coping styles and anxiety in college students, it was found that negative coping styles were a major predictor of anxiety (Mahmoud et al., 2012). Anxiety is a state caused by an individual's perception of and reaction to stressful events, which is not only affected by impact of event but also by coping style (Beck and Clark, 1997). Therefore, it can be hypothesized that there may be a chain-mediated relationship between coping style and anxiety in the path from impact of event to FoMO.

### 1.5 Aims

In summary, college students may face challenges such as failed exams and strained relationships in both their studies and personal lives. If these challenges are not addressed rationally, negative emotions such as anxiety may arise. Additionally, students are often heavily involved in social media and mobile phone networks, which can contribute to FoMO. Therefore, this study examines the relationship between impact of event, coping style, anxiety, and FoMO. The analysis focuses on the impact of event on FoMO and coping style, as well as anxiety. Three dimensions of an event impact will be discussed in separate models.

### 1.6 Hypothesis of the current study

**Figure 1:**
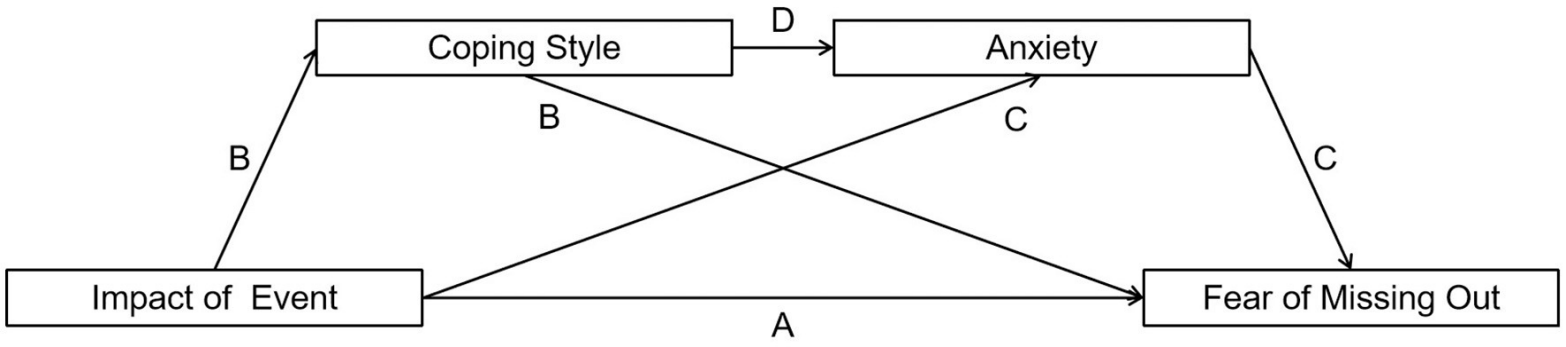
Full proposed model for the mediating role of coping style and anxiety on the association between impact of event and the fear of missing out. The full proposed model included: **(A)** The direct effect from impact of event to fear of missing out; **(B)** The mediating role of coping style in the path from impact of event to fear of missing out; **(C)** The mediating role of anxiety in the path from impact of event to fear of missing out; and **(D)** The path from coping style to anxiety.

Based on the above arguments, the current study suggests that the impact of event affected FoMO through the chain mediation effect of coping style and anxiety. Therefore, the following research hypothesis is proposed (see Figure 1 for the full illustration of the full model): H1: Impact of event can positively predict FoMO. H2: Coping style mediates the path from impact of event to FoMO. H3: Anxiety mediates the path from impact of event to FoMO. H4: Coping style negatively predict anxiety.

## 2 Method

### 2.1 Participants and procedure

Participants were selected based on the criteria of being college students aged 19–24 in Guangdong Province, excluding those with known cognitive impairments or psychiatric disorders. A total of 1143 questionnaires were collected, the questionnaire was considered invalid if more than 3 questions were omitted or if the same option was selected more than 20 times in a row. Based on this screening criterion, 129 samples were excluded from the analyses. The final sample consisted of 1,014 valid questionnaires, including 367 male students (36.19%) and 647 female students (63.81%), with a mean age of 20.35 years (SD = 1.15, range: 19–24 years). The sample consisted mainly of university grade 1 (*n* = 410, 35.12% male, mean age:19.24 ± 0.44years, range: 19-21years), grade 2 (*n* = 238, 35.29% male, mean age: 20.57 ± 0.82 years, range: 19–23 year) and grade 3 (*n* = 366, 37.97% male, mean age: 21.47 ± 0.61years, range: 21–24 year) students (see Table 1). The study received approval from the Research Ethics Committee of Guangzhou Medical University(permission number: 202310002). Informed consent forms, detailing the study's purpose, the voluntary basis of participation, and the commitment to data privacy, were provided to both the students and their parents. Participation in the study was contingent upon receiving signed consent from the student. Those who consented were then guided to complete the questionnaires in a supervised setting within their schools.

**Table 1:** Demographic information.

**Characteristics**	**Sample size (*n* male)**	**Mean age (*SD*)**
Total sample	1014 (367)	20.35 (1.15)
**Grade**		
Grade 1	410 (144)	19.24 (0.44)
Grade 2	238 (84)	20.57 (0.82)
Grade 3	366 (139)	21.47 (0.61)

### 2.2 Measures

#### 2.2.1 Impact of event

Impact of event was measured by the Chinese version (Guo et al., 2007) of the Impact of Event Scale-Revised (IES-R). Three dimensions including avoidance (e.g., “I tried to remove it from my memory.”), intrusion (e.g., “I found myself acting or feeling like I was back at that time.”), and hyperarousal (e.g. “I had trouble concentrating.”) were included in the scale. Respondents were asked to rate 22 items (8 items for avoidance, 8 items for intrusion, and 6 items for hyperarousal) on a five-point Likert scale from 1 (Not at all) to 5 (Extremely). The total score for each dimension was calculated to create composite scores for avoidance impact of event, intrusion impact of event, and hyperarousal impact of event separately. Higher scores always represent higher levels of the impact of event. In this study, the Cronbach's alpha coefficient of the IES-R was 0.933 (0.837 for avoidance subscale, 0.878 for intrusion subscale, and 0.804 for hyperarousal subscale).

#### 2.2.2 Coping style

Coping style was measured by the Simplified Coping Style Questionnaire (SCSQ) (Xie, 1998). The SCSQ comprises 20 items, assessing participants' positive coping style (12 items, e.g., “I will try to see the good side of things.”) and negative coping style (8 items, e.g., “I will smoke, drink, take drugs and eat too much to relieve my worries.”). Respondents were asked to rate each item on a four-point Likert scale from 1 (Not using this strategy) to 4 (This strategy is often used). The total score of the positive coping style subscale was subtracted from the total score of the negative coping style subscale to obtain a composite score of coping style, with larger composite scores indicating a greater tendency for individuals to use positive coping styles. The Cronbach's alpha coefficient for the scale in this study was 0.865 (0.865 for positive coping style subscale and 0.721 for negative coping style subscale).

#### 2.2.3 Anxiety

The Chinese version of the anxiety subscale from the Symptom Checklist-90 (SCL-90) (Derogatis et al., 1976). was used in the current study. This subscale comprises 10 items, with examples such as “Suddenly scared for no reason” and “Spells of terror or panic.” Each item was rated on a five-point Likert scale from 1 (Not at all) to 5 (Extremely). The total score across all items were calculated, with higher scores indicating higher levels of anxiety. In the present study, the Cronbach's alpha coefficient for the anxiety subscale from SCL-90 was 0.882.

#### 2.2.4 Fear of missing out

The Fear of Missing Out Scale (Przybylski et al., 2013) was used to access the pervasive apprehension that others might be having rewarding experiences from which one is absent. Respondents were requested to rate 10 items on a five-point scale from 1 (Not at all true of me) to 5 (Extremely true of me). Examples include “I fear others have more rewarding experiences than me” and “I get anxious when I don't know what my friends are up to.” The scores were summed, with a larger score indicating more fear of missing out. The Cronbach's alpha coefficient of the Fear of Missing Out Scale was 0.878 in this study.

### 2.3 Statistical approach

The mediating effects were estimated following the structural equation modeling procedures using Mplus 7.0. Bootstrap procedures were used to test and validate the statistical significance of the paths. Model fit was assessed using a variety of fit indices, including the ratio chi-square over degrees of freedom (λ^2^/*df*), comparative fit index (CFI), root mean square error of approximation (RMSEA), standardized root mean square residual (SRMR), and Tucker-Lewis index (TLI). Previous literature (Hoyle, 2012) indicates that the model fit is good when λ^2^/*df* ≤ 5; *CFI* ≥ 0.95, *TLI* ≥ 0.95, *RMSEA* ≤ 0.06, and *SRMR* ≤ 0.08. The bootstrapping procedure computes an estimation of the indirect effect with a 95% confidence interval (CI). The indirect effect is considered significant when zero is excluded from the confidence interval.

## 3 Results

### 3.1 Descriptive statistics

Means, standard deviations, and correlations across all variables are presented in Table 2. The scores of the three subscales (avoidance, intrusion, and hyperarousal) from the IES-R were positively correlated with the indicator of the FoMO (*r*_1_ = 0.15, *r*_2_ = 0.16, *r*_3_ = 0.17; *ps* < 0.001), indicating that a greater impact of the event leads to more FoMO. Coping style was negatively correlated with the scores of the avoidance and the hyperarousal subscale from the IES-R (*r*_1_ = −0.07, *r*_2_ = −0.09; *ps* < 0.05), while anxiety was positively correlated with the scores of the three subscales from the IES-R (*r*_1_ = 0.19, *r*_2_ = 0.17, *r*_3_ = 0.25; *ps* < 0.001).

**Table 2:** Descriptive statistics and correlations for all variables.

**Variables**	**M**	**SD**	**1**	**2**	**3**	**4**	**5**	**6**
1. Impact of event: Avoidance	12.96	4.29	1					
2. Impact of event: Intrusion	13.91	4.71	–0.75***	1				
3. Impact of event: Hyperarousal	9.85	3.61	0.73***	0.73***	1			
4. Coping style	13.9	6.49	–0.07*	0.01	–0.09**	1		
5. Anxiety	12.74	4.35	0.19***	0.17***	0.25***	–0.12***	1	
6. Fear of missing out	26.2	7.22	0.15***	0.16***	0.17***	–0.01	0.29***	1

Impact of event are reported in three dimensions (i.e., avoidance, intrusion, and hyperarousal), with M referring to the mean and SD to standard deviation. ***p < 0.01, **p < 0.01, *p < 0.05.

### 3.2 The mediating role of coping style and anxiety on the association between avoidance impact of event and the FoMO

**Figure 2:**
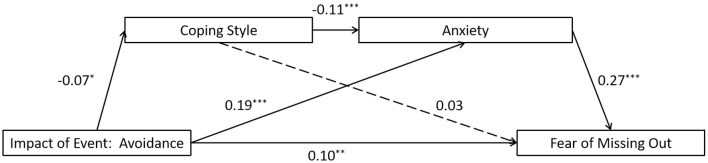
Results of the full proposed model for the mediating role of coping style and anxiety on the association between avoidance impact of event and the fear of missing out. Significant standardized paths are displayed by the solid line. Paths that did not reach the significance level are indicated by dashed lines. ^***^p < 0.001, ^**^p < 0.01, ^*^p < 0.05.

**Figure 3:**
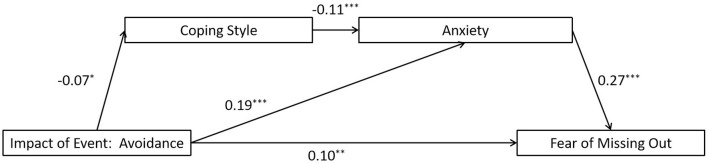
Results of the alternative model for the mediating role of coping style and anxiety on the association between avoidance impact of event and the fear of missing out. Significant standardized paths are displayed by the solid line. Paths that did not reach the significance level are indicated by dashed lines. ^***^p < 0.001, ^**^p < 0.01, ^*^p < 0.05.

The full proposed model was constructed, including: (a) the direct path: avoidance impact of event → the FoMO, (b) the indirect path for coping style: avoidance impact of event → coping style → the FoMO, (c) the indirect path for anxiety: avoidance impact of event → anxiety → the FoMO, (d) the direct path for mediators: coping style → anxiety. The path from coping style to the FoMO did not reach significant level in the full proposed model (see Figure 2 for standardized path coefficients). The full proposed model was a saturated model (λ^2^(*df* = 0) = 0; *CFI* = 1.000, *TLI* = 1.000, *SRMR* = 0.000, and *RMSEA* = 0.000). Consequently, we eliminated the path between them (coping style → the FoMO) to construct an alternative model, and the model fit indices [λ^2^(*df* = 1) = 1.226,*p* = 0.268; CFI = 0.998, TLI = 0.991, SRMR = 0.009, and RMSEA = 0.015] were within an acceptable range (see Figure 3 for standardized path coefficients). The alternative model fits the data well and is more concise compared to the full model. Therefore, the alternative model was selected as the final model. The indirect effects for the full model are reported in Table 3. Bootstrapping analyses suggested that the mediating effects of anxiety alone and the chain mediation effect of coping style and anxiety reached a significant level (indirect effects = 0.050, 0.001, respectively; *ps* < 0.05).

**Table 3:** Indirect effects for the mediating role of coping style and anxiety on the association between hyperarousal impact of event and the fear of missing out.

**Path**	***b***	**95% CI**
Avoidance impact of event → coping style → the fear of missing out	–0.002	–0.007, 0.002
Avoidance impact of event → anxiety → the fear of missing out	0.05	0.033, 0.067
Avoidance impact of event → coping style → anxiety → the fear of missing out	0.001	0.001, 0.003

Parameter *b* referring to the standardized path coefficient, CI referring to the confidence intervals. The 95% CI was estimated by a Bootstrap procedure.

### 3.3 The mediating role of coping style and anxiety on the association between intrusion impact of event and the FoMO

**Figure 4:**
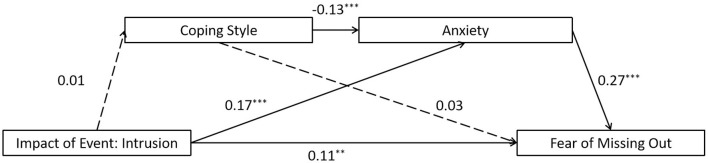
Results of the full proposed model for the mediating role of coping style and anxiety on the association between intrusion impact of event and the fear of missing out. Significant standardized paths are displayed by the solid line. Paths that did not reach the significance level are indicated by dashed lines. ^***^p < 0.001, ^**^p < 0.01.

**Figure 5:**
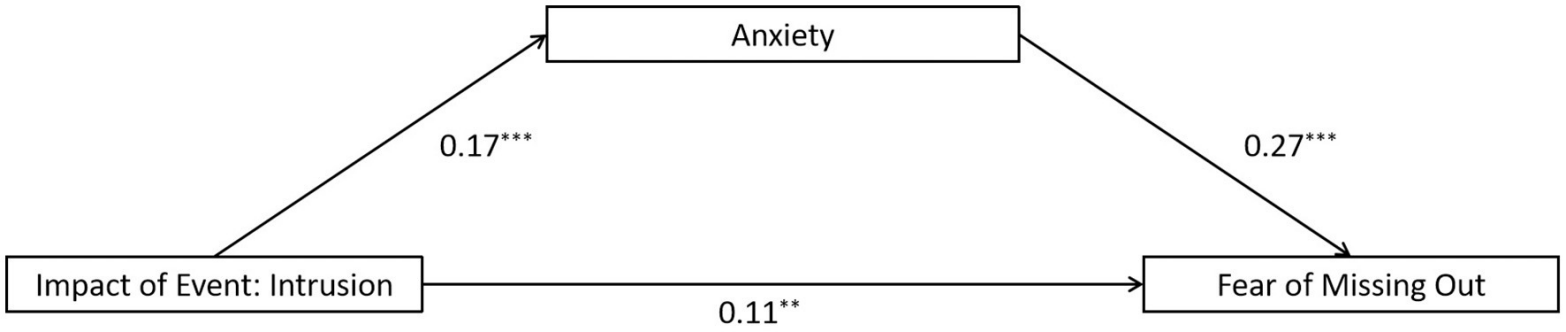
Results of the alternative model for the mediating role of coping style and anxiety on the association between intrusion impact of event and the fear of missing out. Significant standardized paths are displayed by the solid line. Paths that did not reach the significance level are indicated by dashed lines. ^***^p < 0.001, ^**^p < 0.01.

The full proposed model was constructed, including: (a) the direct path: intrusion impact of event → the FoMO, (b) the indirect path for coping style: intrusion impact of event → coping style → the FoMO, (c) the indirect path for anxiety: intrusion impact of event → anxiety → the FoMO, (d) the direct path for mediators: coping style → anxiety. The full proposed model was a saturated model (λ^2^(*df* = 0) = 0; *CFI* = 1.000, *TLI* = 1.000, *SRMR* = 0.000, and *RMSEA* = 0.000). The path from intrusion impact of event to coping style and the path from coping style to the FoMO did not reach a significant level in the full proposed model (see Figure 4 for standardized path coefficients), indicating that the coping style did not show mediation effect in this model. Therefore, we removed the coping style from the full model and construct an alternative model. As the alternative model is a saturated model (λ^2^(*df* = 0) = 0; *CFI* = 1.000, *TLI* = 1.000, *SRMR* = 0.000, and *RMSEA* = 0.000). Nevertheless, the alternative model is more concise compared to the full model (see Figure 5 for standardized path coefficients). Therefore, the alternative model was selected as the final model. The indirect effects for the full model are reported in Table 4. Bootstrapping analyses suggested that the mediating effects of anxiety alone reached significant level (indirect effect = 0.046, *ps* < 0.05).

**Table 4:** Indirect effects for the mediating role of coping style and anxiety on the association between intrusion impact of event and the fear of missing out.

**Path**	***b***	**95% CI**
Intrusion impact of event → coping style → the fear of missing out	0	–0.002, 0.003
Intrusion impact of event → anxiety → the fear of missing out	0.046	0.030, 0.062
Intrusion impact of event → coping style → anxiety → the fear of missing out	0	–0.002, 0.002

Parameter *b* referring to the standardized path coefficient, CI referring to the confidence intervals. The 95% CI was estimated by a Bootstrap procedure.

### 3.4 The mediating role of coping style and anxiety on the association between hyperarousal impact of event and the FoMO

**Figure 6:**
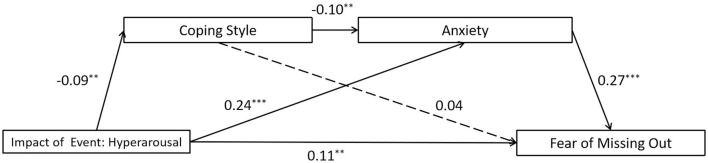
Results of the full proposed model for the mediating role of coping style and anxiety on the association between hyperarousal impact of event and the fear of missing out. Significant standardized paths are displayed by the solid line. Paths that did not reach the significance level are indicated by dashed lines. ^***^p < 0.001, ^**^p < 0.01.

**Figure 7:**
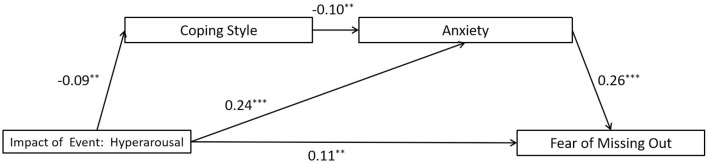
Results of the alternative model for the mediating role of coping style and anxiety on the association between hyperarousal impact of event and the fear of missing out. Significant standardized paths are displayed by the solid line. Paths that did not reach the significance level are indicated by dashed lines. ^***^p < 0.001, ^**^p < 0.01.

The full proposed model was constructed, including: (a) the direct path: hyperarousal impact of event → the FoMO, (b) the indirect path for coping style: hyperarousal impact of event → coping style → the FoMO, (c) the indirect path for anxiety: hyperarousal impact of event → anxiety → the FoMO, (d) the direct path for mediators: coping style → anxiety. The full proposed model was a saturated model [λ^2^(*df* = 0) = 0; *CFI* = 1.000, *TLI* = 1.000, *SRMR* = 0.000, and *RMSEA* = 0.000]. The path from coping style to the FoMO did not reach a significant level in the full proposed model (see Figure 6 for standardized path coefficients). Therefore, we deleted the path between them (coping style → the FoMO) to construct an alternative model, and the model fit indices (λ^2^(*df* = 1) = 1.386, *p* = 0.239; CFI = 0.998, TLI = 0.987, SRMR = 0.009, and RMSEA = 0.020) were within an acceptable range (see Figure 7 for standardized path coefficients). The alternative model fits the data well and is more concise compared to the full model. Therefore, the alternative model was selected as the final model. The indirect effects for the full model are reported in Table 5. Bootstrapping analyses suggested that the mediating effects of anxiety alone and the chain mediation effect of coping style and anxiety reached significant level (indirect effects = 0.062, 0.002, respectively; *ps* < 0.05).

**Table 5:** Indirect effects for the mediating role of coping style and anxiety on the association between hyperarousal impact of event and the fear of missing out.

**Path**	***b***	**95% CI**
Hyperarousal impact of event → coping style → the fear of missing out	–0.003	–0.009, 0.002
Hyperarousal impact of event → anxiety → the fear of missing out	0.062	0.045, 0.079
Hyperarousal impact of event → coping style → anxiety → the fear of missing out	0.002	0.001, 0.005

Parameter *b* referring to the standardized path coefficient, CI referring to the confidence intervals. The 95% CI was estimated by a Bootstrap procedure.

## 4 Discussion

This study investigates the various factors contributing to FoMO, diverging from previous studies that focused on the negative outcomes associated with FoMO. By collecting samples from a population of college students, the present study analyzed the association between impact of event and FoMO, and investigated how the relationship was influenced by coping style and anxiety. A multiple mediator model revealed a positive association between the impact of event and FoMO levels in college students, thus supporting H1. Coping style and anxiety were identified as chain mediators in the pathway from impact of event to FoMO, thus supporting H2 and H3.

The results revealed a positive correlation between the impact of event and the level of FoMO, indicating that individuals who are more affected by major life events tend to experience more severe FoMO symptoms. This finding supports H1 and aligns with explanations from self-regulation theory. The impact of event disrupts the intended and cyclical self-regulatory processes, triggering self-regulatory defense mechanisms, which ultimately leads to a blockage of self-regulation (Zimmerman, 2000; Pintrich et al., 2012). With a greater impact, the degree of diffuse anxiety stemming from blocked self-regulation worsens, ultimately exacerbating FoMO.

A noteworthy discovery of this study is that all three impact of event dimensions positively predicted FoMO. However, they did not follow identical paths in the models. A chain of mediation effect of coping style and anxiety in the path from the avoidance factor and the hyperarousal factor to FoMO was found. While the mediating effect of coping style did not reach significant level in the path from the intrusion factor to FoMO. The mediating effect of anxiety remained significant cross all models.

Previous discussions on the impact of event on FoMO have tended to focus on life events as a distinct structure (Garnefski et al., 2001; McLaughlin and Hatzenbuehler, 2009; Turkle, 2011; Davies et al., 2022). The variability of individuals' post-event psychological responses, as measured by the dimensions of intrusion, hyperarousal, and avoidance, implies that not all individuals experience the event in the same way. As a result, their coping styles and psychological states may also vary post-event.

Impact of event can induce high levels of arousal in an individual's physiological and psychological state, which can hinder self-regulation. The pressure to regulate the situation can lead to over-defense, negative coping mechanisms, anxiety, and subsequently, FoMO. Due to the intense pain brought on by the impact of the event, the defense mechanism is triggered, and avoidance strategies are employed (Pintrich et al., 2012). However, simply avoiding the situation does not eliminate the pain caused by the impact of event. Avoidance is a negative coping mechanism that only provides short-term relief from perceived danger and pain. This can prevent cognitive change and maintain cognitive dissonance. Negative coping mechanisms can also increases anxiety and leads to FoMO (Mahmoud et al., 2012; Roohafza et al., 2014). In contrast to the dimensions of avoidance and heightened arousal, the intrusive dimension involves abnormal activation of the individual's memory system. This leads to uncontrollable and repeated recall and experience of fragments, images, and sensations associated with the impact of event. Event-related memories are abnormally reinforced and consolidated, resulting in their frequent and involuntary intrusions into the individual's consciousness, such as nightmares due to intrusive memories. Intrusions are recollections of past events that remain at the conscious level and do not affect behavior. Therefore, they do not impact coping style. This could be a possible explanation for the lack of significance in the relationship between coping style and the intrusion dimension. However, the impact of event was intrusive and caused the individual to experience negative emotions continuously, leading to anxiety and triggering their FoMO.

The degree of positive coping style was significantly and negatively related to the level of anxiety, supporting H4 and consistent with the findings of previous studies (Kozora et al., 2005; Lewis et al., 2012). The impact of event places tremendous external pressure on the individual, making it difficult for the cognitive system to reconcile the internal and external environments into rational coping styles, resulting in poor coping styles (Lazarus, 1974; Folkman and Lazarus, 1988). Poor coping styles do not solve the problems associated with the impact of event in life but instead increase the individual's negative emotions, causing anxiety and triggering FoMO (Beck and Clark, 1997). The current results showed that the greater the impact of the event, the more negative the coping style of an individual under stress. The negative coping style leads to an increase in the level of anxiety, which further exacerbates FoMO. Therefore, there is a chain mediating effect between coping style and anxiety in the impact of the event to FoMO pathway.

We explored the path from the impact of event to FoMO, concerning three factors of the impact of event. This study provides a deeper understanding of the origins and mechanisms behind FoMO, thereby providing a theoretical basis for interventions to address this phenomenon. The results revealed that the anxiety caused by the impact of event will make college students prone to FoMO, when confronted with the impact of event, it is important to avoid long-term and high-arousal negative emotions using positive coping strategy.

This study has several limitations: (1) the cross-sectional study design limits the inference of causal relationships between variables; future studies should employ longitudinal studies to more accurately explain causal relationships between variables; (2) data were collected through self-reporting in a retrospective manner, and the accuracy of the data is influenced by individual subjectivity; future studies could incorporate behavioral tasks to measure FoMO, anxiety, and other variables; (3) there may be multiple types of FoMO (Abri, 2017; Wegmann et al., 2017), and the influencing factors of different types of FoMO and their mechanisms should be addressed in further research.

## 5 Conclusion

This study explored the relationship between impact of event on FoMO and provided a more comprehensive reference basis for FoMO intervention by further clarifying the psychological factors and mechanisms affecting FoMO. The results of the study showed that (1) the impact of event is positively correlated with FoMO, indicating that the more an individual is affected by life events, the more severe the FoMO symptoms; (2) the positive degree of coping style is negatively correlated with the degree of anxiety (3) there is a chain mediation between coping style and anxiety in the hyperarousal and avoidance factor of the impact of event to the FoMO; (4) anxiety plays a mediates role in the link between the impact of event and FoMO.

## Data availability statement

The raw data supporting the conclusions of this article will be made available by the authors, without undue reservation.

## Ethics statement

The study was approved by the Research Ethics Committee of Guangzhou Medical University (#202310002). The studies were conducted in accordance with the local legislation and institutional requirements. The participants provided their written informed consent to participate in this study.

## Author contributions

BH: Conceptualization, Data curation, Formal analysis, Methodology, Writing – original draft, Writing – review & editing. ZT: Conceptualization, Data curation, Writing – review & editing, Formal analysis. KL: Conceptualization, Writing – review & editing. BQ: Conceptualization, Data curation, Writing – review & editing. SW: Conceptualization, Methodology, Writing – review & editing.
